# Sinonasal and Orbital Imaging Findings in COVID-Associated Rhino-Orbito-Cerebral Mucormycosis During the Second Wave of COVID-19: A Retrospective Cohort Study in a Tertiary Hospital in Central India

**DOI:** 10.7759/cureus.42674

**Published:** 2023-07-30

**Authors:** Shrikrishna B H, Sunita Kumbhalkar, Kalai Selvi, Deepa G, Vijay Bidkar, Sandeep Dabhekar, Kirankumar Prathipati, Anupama Sawal

**Affiliations:** 1 Otolaryngology - Head and Neck Surgery, All India Institute of Medical Sciences, Bibinagar, Hyderabad, IND; 2 General Medicine, All India Institute of Medical Sciences, Nagpur, Nagpur, IND; 3 Community Medicine, All India Institute of Medical Sciences, Nagpur, Nagpur, IND; 4 Anatomy, Datta Meghe Medical College, Nagpur, IND; 5 Otolaryngology - Head and Neck Surgery, All India Institute of Medical Sciences, Nagpur, Nagpur, IND; 6 Anatomy, Jawaharlal Nehru Medical College, Datta Meghe Institute of Higher Education and Research, Wardha, IND

**Keywords:** computed tomography (ct), paranasal sinus diseases, covid-19, imaging findings, rhino-orbito-cerebral mucormycosis

## Abstract

Background

Mucormycosis is a consequence of the angioinvasive disease caused by filamentous fungi that belong to the order Mucorales, particularly *Mucor*, *Rhizopus*, and *Rhizomucor*. *Rhizopus oryzae* is the most prevalent form. The invading hyphae lead to damage of blood vessels leading to thrombosis and consequent tissue necrosis. The incidence of this disease entity witnessed a significant rise during the second wave of the coronavirus disease 2019 (COVID-19) pandemic. Timely diagnosis and prompt treatment are crucial to diminish both the mortality and morbidity associated with this disease. Imaging plays a pivotal role in diagnosing the ailment, evaluating its extent, identifying complications such as thrombosis, and facilitating surgical planning. It demonstrates exceptional sensitivity in detecting the disease at its early stages, often before symptoms manifest. Due to the angioinvasive nature of *Mucor*, early detection assumes utmost importance as it necessitates intensive antifungal therapy and the removal of devitalized tissue through debridement.

Methodology

We conducted a retrospective cohort study to analyze computed tomography (CT) imaging findings in patients with COVID-associated rhino-orbito-cerebral mucormycosis (ROCM) confirmed by histopathological examination. We compared these findings with CT findings of the nose and paranasal sinuses in patients without mucor following COVID-19 sinusitis (non-ROCM).

Results

All 16 cases in the non-ROCM group were in stage 1 disease. In contrast, in the ROCM group, three patients had stage 1 disease, five patients had stage 2 disease, and 10 patients had stage 3 disease (p = 0.0001). The pterygopalatine fossa was significantly affected in 10 of 18 ROCM patients and in none of the non-ROCM patients.

Conclusions

Imaging plays a crucial role in the early detection of mucormycosis. It assists treating physicians in initiating prompt and aggressive treatment, thereby improving the prognosis of this frequently fatal disease.

## Introduction

Angioinvasive mucormycosis is a disease that primarily affects individuals with compromised immune systems. The incidence of these cases increased significantly during the second wave of the coronavirus disease 2019 (COVID-19) pandemic due to the disease itself and its treatment weakening the immune systems of patients. Timely diagnosis and appropriate medical therapy are critical to reduce the morbidity and mortality associated with this disease. Utilizing imaging techniques enables the diagnosis of the disease, determination of its extent, detection of complications such as thrombosis, and facilitates surgical planning. It exhibits a high sensitivity for early detection, even before the onset of symptoms.

To effectively decrease the morbidity and mortality of mucormycosis, early detection and consistent treatment are paramount. However, there are limited studies available on the imaging findings in COVID-19-associated rhino-orbito-cerebral mucormycosis (ROCM). In this retrospective cohort study, our objective is to investigate the sinonasal and orbital imaging findings in COVID-19-associated ROCM. We reviewed the clinical records and conducted a comprehensive analysis of the sinonasal imaging patterns of patients with COVID-19-associated sinusitis and ROCM admitted to the ENT department of AIIMS Nagpur from May 2021 to September 2021. We hypothesize that patients with COVID-19-associated ROCM would exhibit more pronounced bone erosion and destruction compared to those without ROCM. Our primary goals were to describe the pattern of nasal and sinus involvement in COVID-19-associated ROCM and to compare the extent of nasal and sinus involvement, as assessed by radiological features, between patients with histopathological evidence (HPE)-positive and HPE-negative features who presented with sinusitis and were admitted to the tertiary care facility after COVID-19.

## Materials and methods

Our study conducted at the All India Institute of Medical Sciences, Nagpur was a retrospective cohort analysis with a duration of six months. The data source consisted of patients admitted to our hospital during the second wave of COVID-19 in India, who were diagnosed with sinusitis associated with COVID-19. The medical records department of the institute provided access to the necessary medical records. The sample size for our retrospective cohort study was determined based on the hospital records. After obtaining approval from the institution’s ethics committee, all cases admitted to the ENT department with a diagnosis of sinusitis and confirmed COVID-19 infection between May 1, 2021, and October 30, 2021, were included in the study. There was no selection bias as all cases meeting the inclusion criteria during the specified period were selected. The inclusion criteria encompassed patients who had tested positive for COVID-19 within six months before the admission date and displayed CT evidence of acute sinusitis during their visit to the ENT outpatient department. Image density and signal strength were assessed, and the non-enhanced CT density images were scored and compared with brain or muscle images.

A suspected case of ROCM is characterized by clinical findings suggestive of mucormycosis and a paranasal CT scan that indicates a diagnosis of mucormycosis. A confirmed case is determined when clinical findings of mucormycosis are accompanied by a histopathological diagnosis obtained through an endoscopic biopsy. COVID-19-associated ROCM is defined as a suspected or confirmed case of ROCM meeting the aforementioned criteria, along with a positive reverse transcription polymerase chain reaction (RT-PCR) report for COVID-19 within the past six months from the onset of symptoms. COVID-19-associated non-ROCM sinusitis, on the other hand, is defined as a case of acute sinusitis confirmed based on clinical and CT evidence, with a positive RT-PCR report for COVID-19 within the past six months from the onset of symptoms, and with histopathology ruling out *Mucor *infection. Subjects were enrolled in either the mucormycosis group or the non-mucormycosis group based on the histopathological report of the nasal biopsy specimen obtained during sinus surgery during their in-patient stay.

The inclusion criteria for the COVID-19-associated mucormycosis group were as follows: (1) positive RT-PCR report for COVID-19 within the past six months from the onset of sinusitis symptoms, (2) confirmation of sinusitis through CT of the paranasal sinuses, and (3) confirmation of mucormycosis through a histopathology report of nasal tissue. The exclusion criterion for the COVID-19-associated mucormycosis group was the presence of a histopathology report ruling out mucormycosis in nasal tissue.

For the COVID-19-associated non-mucormycosis group, the inclusion criteria were as follows: (1) positive RT-PCR report for COVID-19 within the past six months from the onset of sinusitis symptoms, (2) confirmation of sinusitis through CT of the paranasal sinuses, and (3) mucormycosis ruled out through a histopathology report of nasal tissue. The exclusion criterion for the COVID-19-associated non-mucormycosis group was the presence of a histopathology report confirming mucormycosis in nasal tissue.

We examined the medical records of individuals belonging to the two groups to analyze the pattern of nose and sinus involvement. The extent of nose and sinus involvement was assessed in both groups and compared using appropriate statistical methods. Patient characteristics were summarized in terms of frequencies. The various patterns of radiological imaging findings, such as sinus opacification, sinonasal bony wall erosion, air-fluid level, and non-enhancement, were also summarized in terms of frequencies and percentages, accompanied by a 95% confidence interval. The association between specific imaging findings in confirmed ROCM and non-ROCM cases was presented as relative risk with a 95% confidence interval. Statistical tests, including t-test, chi-square test, and non-parametric tests, were employed during the analysis.

Following the categorization proposed by Rupa et al. [1], the cases were classified into three categories as follows: Stage 1: involvement limited to the nose and paranasal sinuses; Stage 2: extensive spread to the orbit, palate, or oral cavity; and Stage 3: severe disease with invasion of the intracranial, periorbital, or pterygopalatine fossa.

## Results

A total of 34 post-COVID-19 patients were admitted during our study period with the presentation of sinusitis. Overall, 18 patients had histopathological confirmation of mucormycosis and were included in the ROCM group. Further, 16 patients were negative for mucormycosis according to histopathology and were included in the non-ROCM group. The mean age in the ROCM group was 55.8 years (SD = 14.3) and in the non-ROCM group was 50.1 years (SD = 11.7) (p = 0.22). In the ROCM group, 11 were males and seven were females. In the non-ROCM group, eight were males and eight were females (p = 0.52). In the ROCM group, 12 were known diabetics on treatment. In the non-ROCM group, only six were known diabetics on treatment (p = 0.09). In the ROCM group, at the time of the presentation, the random blood sugar level was 183.2 mg%. In the non-ROCM group, at the time of the presentation, the random blood sugar level was 143.9 mg% (p = 0.23, non-parametric). In the ROCM group, the HbA1C level at presentation was 7.2. In the Non-ROCM group, the HbA1C level at presentation was 6.7 (p = 0.57) (Table 1).

**Table 1:** Comparison of demographic and clinical characteristics of patients between confirmed Mucor (ROCM) and non-Mucor (non-ROCM) sinus involvement (n = 34). *: non-parametric. ROCM: rhino-orbito-cerebral mucormycosis

Characteristics	ROCM (n = 18)	Non-ROCM (n = 16)	P-value
Age (in years), mean (SD)	55.8 (14.3)	50.1 (11.7)	0.22
Sex			
Male	11 (57.9)	8 (42.1)	
Female	7 (46.7)	8 (53.3)	0.52
History of diabetes			
Absent	6 (37.5)	10 (62.5)	
Present	12 (66.7)	6 (33.3)	0.09
Blood sugar (mg%)	183.2 (93.2)	143.9 (46.4)	0.23*
HbA1C	7.1 (2.0)	6.7 (1.5)	0.57

The nasal cavity floor was significantly involved in seven patients in the ROCM group but none in the non-ROCM group (p = 0.008). The lateral wall of the nose was significantly involved in 16 patients in the ROCM group but none in the non-ROCM group (p = 0.0001). The paranasal sinuses bone was significantly involved in nine patients in the ROCM group and two patients in the non-ROCM group (p = 0.03). Isolated maxillary bone erosion was seen in one case in the ROCM group but in five cases in the non-ROCM group (p = 0.08). Isolated sphenoid sinus bone erosion was seen in one case in the ROCM group and none in the non-ROCM group (p = 0.9). Involvement of both maxillary and ethmoids was noted in three patients in the ROCM group and five patients in the non-ROCM group (p = 0.43). Involvement of sphenoids, maxillary, and ethmoids was noted in two patients in the ROCM group and three patients in the non-ROCM group (p = 0.65). Involvement of frontal, maxillary, and ethmoids was noted in two patients in the ROCM group and one patient in the non-ROCM group (p = 0.62). Bilateral sinus bones were involved in five patients in the ROCM group and five patients in the non-ROCM group (p = 1.0). The hard palate was significantly involved in eight patients in the ROCM group and none in the non-ROCM group (p = 0.003). Zygoma was involved in four patients in the ROCM group and none in the non-ROCM group (p = 0.11). The pre-maxillary area was significantly involved in nine patients in the ROCM group and none in the non-ROCM group (p = 0.001). The retromaxillary area was significantly involved in 11 patients in the ROCM group and none in the non-ROCM group (p = 0.0001). Pterygomaxillary fossa was significantly involved in 10 patients in the ROCM group and none in the non-ROCM group (p = 0.0001). Pterygopalatine bone involvement was significantly observed in eight patients in the ROCM group and none in the non-ROCM group (p = 0.003). Infratemporal fossa was involved in three patients in the ROCM group and none in the non-ROCM group (p = 0.23). The medial wall of the orbit was significantly involved in seven patients in the ROCM group and none in the non-ROCM group (p = 0.008). The floor of orbit was involved in four patients in the ROCM group and none in the non-ROCM group (p = 0.11). The lateral wall of the orbit was involved in one patient in the ROCM group and none in the non-ROCM group (p = 1.0). Bilateral orbital bone erosion was significantly observed in 10 patients in the ROCM group and none in the non-ROCM group (p = 0.0001). Intracranial involvement was observed in two patients in the ROCM group and none in the non-ROCM group (p = 0.49) (Table 2).

**Table 2:** Comparison of patterns of paranasal sinus, orbit, and nasal involvement among confirmed ROCM and non-ROCM patients (n = 34). ROCM: rhino-orbito-cerebral mucormycosis

Area involved	ROCM (n = 18)	Non-ROCM (n = 16)	P-value
Nasal involvement			
Nasal floor (n = 7)	7 (100)	0 (0)	0.008
Nasal lateral wall (n = 16)	16 (100)	0 (0)	0.0001
Sinus involvement			
Pansinus involvement (n = 11)	9 (81.8)	2 (18.2)	0.03
Isolated maxillary involvement (n = 6)	1 (16.7)	5 (83.3)	0.08
Isolated sphenoid involvement (n = 1)	1 (100)	0 (0)	0.9
Ethmoid + maxillary (n = 8)	3 (37.5)	5 (62.5)	0.43
Ethmoid + maxillary + sphenoid (n = 5)	2 (40)	3 (60)	0.65
Frontal + maxillary + ethmoid (n = 3)	2 (66.7)	1 (33.3)	0.62
Bilateral sinus involvement (n = 10)	5 (50)	5 (50)	1.0
Hard palate (n = 8)	8 (100)	0 (0)	0.003
Zygoma (n = 4)	4 (100)	0 (0)	0.11
Pre-maxillary (n = 9)	9 (100)	0 (0)	0.001
Retromaxillary (n = 11)	11 (100)	0 (0)	0.0001
Pterygomaxillary fossa	10 (100)	0 (0)	0.0001
Pterygopalatine involvement (n = 8)	8 (100)	0 (0)	0.003
Infratemporal fossa (n = 3)	3 (100)	0 (0)	0.23
Orbit involvement			
Orbit medial wall (n = 7)	7 (100)	0 (0)	0.008
Orbit floor (n = 4)	4 (100)	0 (0)	0.11
Orbit lateral wall (n = 1)	1 (100)	0 (0)	1.0
Bilateral orbit involvement (n = 10)	10 (100)	0 (0)	0.0001
Intracranial involvement (n = 2)	2 (100)	0 (0)	0.49

**Figure 1:**
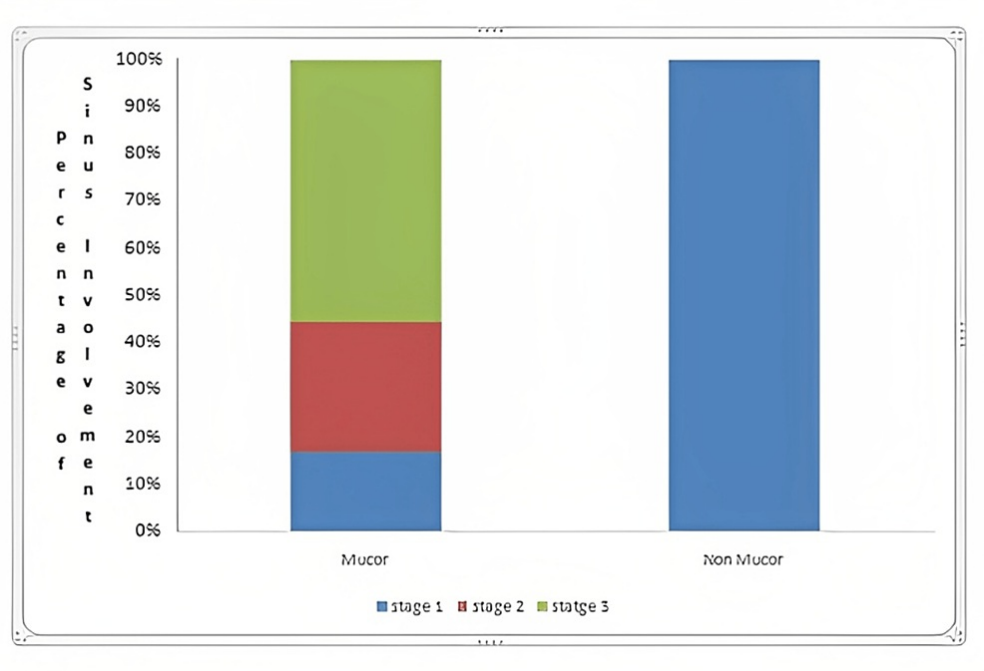
Comparison of tissue involvement stage between confirmed Mucor and non-Mucor cases (n = 34).

In our study, all 16 cases in the non-ROCM group had stage 1 disease. On the other hand, in the ROCM group, three patients had stage 1 disease, five patients had stage 2 disease, and 10 patients had stage 3 disease (p = 0.0001) (Figure 1).

## Discussion

Mucormycosis is an angioinvasive condition that primarily affects individuals with a weakened immune system [2]. The Mucorales order of filamentous mold fungi, which includes the genera *Rhizopus*, *Mucor*, and *Rhizomucor*, is responsible for its development. The disease is transmitted through inhalation of fungal spores, which are widespread in the environment. Regardless of the route of invasion, hyphae destroy tissue by encroaching upon blood vessels [3]. *Rhizopus oryzae* is accountable for roughly 60% of all infections [4]. The disease is transmitted through the inhalation of prevalent fungal spores in the environment. The hyphae of the fungus invade blood vessels, resulting in tissue damage. *Mucor *fungus exhibits a strong affinity for blood vessels, particularly the elastic membrane of arteries, which represents the primary factor underlying its high mortality rate. Upon invading the vascular structures, the spores multiply within the elastic lamina of the arteries. Hyphae erode the endothelium of the vessel walls, leading to subsequent necrosis, thrombi formation, and infarcts [5].

Clinically, mucormycosis manifests in five distinct subtypes, namely, disseminated, cutaneous, gastrointestinal, pulmonary, and rhino-cerebral [6]. The most prevalent subtype, rhino-cerebral mucormycosis, predominantly affects immunocompromised individuals with conditions such as burns, neutropenia, uremia, diabetic ketoacidosis, severe malnutrition, or those undergoing continuous corticosteroid therapy [7-9].

Early diagnosis and effective medical therapy play a critical role in reducing morbidity and mortality associated with the disease [10]. Hyperglycemia or resultant acidosis can disrupt the host’s metabolism, creating an environment conducive to fungal growth. The spores are believed to initially colonize the upper respiratory system, proliferate in the sinuses, and subsequently invade the sinus walls or blood vessels, leading to cerebral involvement [11-13]. 

Imaging plays a crucial role in diagnosing the disease, determining its extent, detecting complications such as thrombosis, and facilitating surgical planning [14]. It exhibits high sensitivity in detecting the disease at its early stages, even before the onset of symptoms. In the appropriate clinical context, the presence of orbital fat stranding and fat stranding in the premaxillary region contribute to early identification on imaging. In cases where mucormycosis is clinically suspected, the initiation of antifungal medication can be supported by imaging data.

Our study revealed a severe sinonasal and orbital inflammatory process in all ROCM patients compared to the non-ROCM group. Similar findings were reported in previous studies by Diego et al. and Mnif et al. [15,16]. Although the aggressive clinical features of the disease may suggest a fungal infection, an accurate diagnosis relies on anatomopathological evidence of tissue invasion by the fungus.

**Figure 2:**
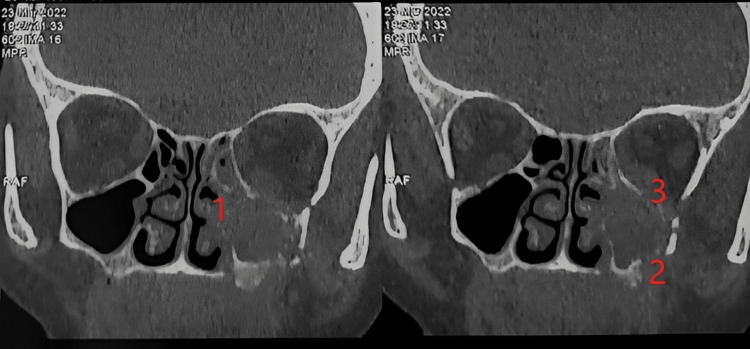
CT image of one of the mucormycosis patients, showing destruction of the medial wall (1), the anterolateral wall (2), and the roof (3) of the left maxillary sinus.

Furthermore, in our study, computed tomography imaging of ROCM revealed a uniform opacification of the sinus cavity with well-defined, notably hyperdense foci within the inflammatory reaction (Figure 2).

Ronald and George highlighted that the presence of uniform mucosal thickening and focal bone damage on imaging often raises suspicion of mucormycosis [17]. According to the authors, the pattern of bone destruction reflects the virulence of the disease. Mucormycosis is characterized by rapid progression and complete bone loss with no apparent cure. When bone destruction is present, mucormycosis is more likely than acute or chronic sinusitis. In cases of chronic pyogenic sinusitis, extensive mucosal thickening is observed instead of bone destruction. Patchy bone degeneration has also been identified as a feature of craniofacial mucormycosis by Green et al. [18]. Similarly, in our study, we also observed bony destruction. To initiate treatment before central nervous system involvement occurs, physicians should maintain a high index of suspicion for mucormycosis, especially in diabetic patients with CT changes suggestive of sinusitis or tumor. Early identification is crucial, and prompt treatment is warranted.

Mucorales fungal infection of the nose and sinuses exhibits rapid spread to the orbit, potentially involving vascular structures such as the internal carotid artery and cavernous sinus. Cerebral involvement in mucormycosis can lead to pathologic manifestations such as purulent meningitis, severe cerebral inflammation, and large infarcts resulting from arterial thrombosis. In addition to the commonly affected frontal lobes, the temporal lobes may also be impacted in this form of disease. CT is the most effective radiographic technique for detecting rhino-cerebral mucormycosis. It accurately reveals the extent of the disease spread to nearby regions such as the infratemporal fossa and pterygomaxillary fissure, as well as the connection between the paranasal sinuses and orbital disease [19]. Hosseini and Borghei suggested that spheno-ethmoidectomy is the only viable treatment for this disease, as the involvement of the orbital apex may contribute to brain and erectile tissue complications [20]. The authors proposed that the primary reservoir for rhino-cerebral mucormycosis is the pterygopalatine fossa, with subsequent spread to the orbit. In our study, significant involvement of the pterygopalatine fossa was observed in 10 out of 18 ROCM patients, while non-ROCM patients did not show such findings. Early detection of clinically asymptomatic lesions and precise disease localization can be achieved through the use of CT imaging.

Following the involvement of the pterygopalatine fossa, the internal maxillary artery and its branches may cause complete necrosis of the maxilla and hard palate [21]. In our study, the hard palate showed significant involvement in eight out of 18 ROCM patients, while none of the non-ROCM patients exhibited such involvement. *Mucor *is able to migrate from the pterygopalatine fossa to the infratemporal fossa through the pterygomaxillary fissure. In our study, the infratemporal fossa was affected in three out of 18 ROCM patients, with no involvement observed in the non-ROCM patients. Despite aggressive drug and surgical treatments, patients with rhino-cerebral mucormycosis that has spread beyond the sinus cavity have a poor prognosis. The presence of skin, palate, and orbit involvement further worsens the prognosis [22]. In our study, all 16 cases in the non-ROCM group exhibited Stage 1 disease. In contrast, among the ROCM group, three patients had Stage 1 disease, five patients had Stage 2 disease, and 10 patients had Stage 3 disease (p = 0.0001).

Although there is a possibility of a cure in treated diabetics and individuals with good health, the infection’s morbidity and the consequences of surgical treatment can have devastating effects on the patient [23]. The ability of CT to detect the disease at an early stage emphasizes its potential value in thoroughly evaluating mucormycosis of the paranasal sinus system. These findings can assist physicians in initiating prompt and aggressive treatment, thus improving the prognosis of this frequently fatal disease.

The main limitation of our study is its small sample size. We acknowledge the limitation of the sample size and the potential impact on the statistical power of the study. Due to the low prevalence of this disease, multinational case-controlled studies are necessary to assess the effectiveness of diagnostic approaches and individual treatment strategies. While we agree that MRI can offer additional insights and sensitivity in detecting certain features of mucormycosis, such as cerebral and orbital involvement, we chose to limit the investigation to CT imaging findings in this retrospective cohort study due to practical constraints and the primary research focus. The inclusion of MRI with contrast and diffusion-weighted imaging was not within the scope of our study, and we acknowledge this limitation.

## Conclusions

Rhino-cerebral mucormycosis typically exhibits rapid progression. Early detection of the condition through CT underscores its potential significance in thoroughly evaluating mucormycosis of the paranasal sinus system. These imaging findings can assist physicians in delivering timely and consistent treatment, thereby enhancing the prognosis of this frequently fatal disease. Debridement of the pterygopalatine fossa, a significant reservoir for *Mucor*, appears to effectively halt disease invasion and have a positive impact on the treatment and prognosis of patients with advanced rhino-cerebral mucormycosis.
